# Bone substitute made from a Brazilian oyster shell functions as a fast stimulator for bone-forming cells in an animal model

**DOI:** 10.1371/journal.pone.0198697

**Published:** 2018-06-05

**Authors:** Ricardo Coringa, Eduardo Martins de Sousa, Juliana Nunes Botelho, Rafael Soares Diniz, Joicy Cortez de Sá, Maria Carmen Fontoura Nogueira da Cruz, Marco Aurelio Beninni Paschoal, Letícia Machado Gonçalves

**Affiliations:** 1 Department of Dentistry, CEUMA University, Sao Luis, Maranhao, Brazil; 2 Department of Immunology and Microbiology of Respiratory Tract Infections, Post-Graduate Program in Parasite Biology, CEUMA University, Sao Luis, Maranhao, Brazil; 3 Department of Oral Rehabilitation, University of Talca, Talca, Chile; 4 Department of Dentistry, Federal University of Maranhao, Sao Luis, Maranhao, Brazil; Universite de Nantes, FRANCE

## Abstract

Despite their demonstrated biocompatibility and osteogenic properties, oyster shells have been reported as a potential alternative to other commonly used materials for bone substitution. This study evaluated whether an experimental bone substitute (EBS) made from a typical oyster shell of Northeastern Brazil (*Crassostrea rhizophora*) has effects on bone development using an animal model. Oysters were collected from a biologically assisted vivarium, and their inner layer was used for preparing an EBS. Chemical and surface characterization of EBS was performed using Individually Coupled Plasma Optical Emission Spectrometry (ICP-OES) and Scanning Electron Microscope (SEM), respectively. Seventy-two rats were randomly assigned to groups according to the treatment of bone defects created in the submandibular area: Negative Control (-C), Positive Control (+C; Bio-Oss^®^) and EBS. Euthanasia occurred at 7, 21, 42 and 56 days postoperatively. The bone pieces were stained with hematoxylin and eosin (H&E). The formation of bone tissue was evaluated histologically and histomorphometrically. Data were analyzed through the Kruskal-Wallis test and ANOVA considering a significant level of 5%. The main element found in EBS was calcium (71.68%), and it presented heterogeneity in the particle size and a porosity aspect at SEM analysis. Histological results revealed the absence of inflammatory cells in all groups, being that EBS presented the most accelerated process of bone formation with a statistically significant difference between this group and the +C and -C groups in the 21-day time-point (*p* < 0.05). After 21 days, the bone formation process was similar between all groups (*p* > 0.05), showing an immature lamellar bone pattern after 56 days of experimentation (*p* > 0.05). Within the limitations of this study, it was possible to conclude that EBS presented good biocompatibility and promoted fast stimulation for bone-forming cells in an animal model.

## Introduction

Implant rehabilitation in partially or totally edentulous patients has become a common practice with predictable long-term results [1–3]. However, edentulism provided by periodontal disease, trauma, malformations or neoplasms can lead to bone atrophies and insufficient bone quantity and/or quality for implant placement [4]. In these cases, ridge augmentation is required to correct the unfavorable bone volume, which can be accomplished with the use of bone grafts and bone-graft substitutes, used individually or combined [2,5–7].

Autogenous bone is considered to be the gold-standard among all bone-grafting material [7,8]. It is highly successful because it contains cellular and matrix components from the same patient that are replaced by newly formed bone, thus providing scaffolding for osteoconduction and growth factors for osteoinduction [8,9]. However, the harvesting procedures could be combined with associated patient morbidity and limitations to harvest reasonable volumes of bone at the time of grafting [10,11].

The bone-graft substitutes serve as a structural scaffold for the attachment of bone marrow cells and other bone-forming cells [6–8,12]. The main groups of bone-graft substitutes are demineralized freeze-dried bone graft, collagen, hydroxyapatite and β-tricalcium phosphate [13–16]. The demineralized freeze-dried bone graft, usually from human or animal donors, is formed by a collagenous matrix and may contain variable amounts of bone morphogenetic proteins (BMPs) [17,18]. It is considered to be osteoinductive, but only osteoconductive in most instances, offering good results besides the high investment necessary [19]. Hydroxyapatite and β-tricalcium phosphate offers the potential for bone substitution because it has a chemical composition close to biological human bone apatite [20]. However, the high reabsorption of this material after grafting is also considered an important limiting factor [21].

Recently, research has demonstrated that nacre obtained from oyster shells presents biocompatibility, biodegradability and osteogenic properties, marking nacre’s potential as an alternative to other commonly utilized biomaterials in tissue engineering [12,21–26]. It is a natural composite material consisting of an inorganic mineral phase and an organic matrix, similar to the structure of human bone. This organized mineral structure is related to its remarkable mechanical strength, while the presence of the organic matrix imparts improved osteoconductivity [23]. The organic matrix has also been found to contain biological molecules practically identical to those found in humans, the BMPs (bone morphogenetic proteins) [27], and other molecules capable of activating osteoblasts through chemical signaling. However, research into these organic molecules has been limited.

Considering that bone-graft substitutes used for osseointegrated implant surgery should ideally have biological properties similar or identical to those of human bones [4,8], nacre derived from oyster shells may be a promising source. Thus, this study aimed to evaluate whether an experimental bone substitute (EBS) made from a typical oyster shell of Northeastern Brazil (*Crassostrea rhizophora*) has effects on bone development using an animal model.

## Materials and methods

### Preparation of EBS

Oyster shells (*C*. *rhizophora*) were collected with the same lifetime, from the same biologically assisted vivarium, from Raposa, Maranhão, Brazil. The oyster shells were cleaned in running water using a steel brush and dried in the sun. The outer surface of the shell was removed with drills under irrigation, resulting in an inner layer free of residues and/or contaminants, also known as the nacre. Using a porcelain hand-held pestle, the nacre was crushed and sifted in a 1.5-mm sieve for particle size standardization. The sieved material was sterilized by gamma irradiation, and the final product was stored in an appropriate sterile vial as EBS.

### Chemical characterization of EBS

Approximately 180.0 mg of EBS were obtained from a sample pool, and this material was subjected in triplicate to the chemical digestion process with 98% sulfuric acid, 65% P.A nitric acid and 30% hydrogen peroxide. After digestion, the sample was stored in a vessel for chemical analysis, which was investigated by Individually Coupled Plasma Optical Emission Spectrometry (ICP-OES).

### Surface characterization of EBS and Bio-Oss®

The microstructure of EBS was assessed using the Scanning Electron Microscope (SEM; JEOL JSM-5600LV; Peabody, MA, USA) to evaluate the size and distribution of particles. The observations were performed on gold-coated specimens and investigated at an acceleration voltage of 15 kV. Bio-Oss® (Geistlich Pharma AG, Wolhusen, Switzerland), a gold-standard demineralized freeze-dried bone graft, was used as a positive control group.

### Animal model

The research proposal was reviewed by the Ethics in Animal Research Committee of CEUMA University, Brazil (Process number 202/2015), and the study design was approved. Seventy-two male, healthy, adult *Wistar* rats weighing 180.0 g on average were used in this study. Prior to surgical procedures, a destress protocol was performed, in which the animals were kept individually in cages for 24 hours. Animals received an intramuscular injection of xylazine chloride (Kensol®; Avellaneda, Argentina; 60mg/Kg body weight) to attain muscular relaxation and were anesthetized intramuscularly with ketamine chloride (Ketalar®; Parke-Davis, Aché Laboratório, São Paulo, SP, Brazil; 0.08mL/100g body weight).

After shaving and asepsis of the submandibular area with 2% chlorhexidine, a 2-cm-long incision was made using a scalpel blade. Skin and periosteal flaps were elevated, the underlying bone tissue was exposed and a defect was prepared with a cylindrical stainless-steel bur (3 mm diameter; 2 mm deep) using a hand piece coupled with a motor for implantation (BML 600; Plus Driller, Sao Paulo, Brazil) at 1,500 rpm under constant sterile saline irrigation, in a sterile environment. Then, animals were randomly assigned to the following groups according to the treatment of the bone defects: Negative Control (-C), Positive Control (+C) and Experimental Group (EBS).

For the Negative Control (-C) group, no bone substitute was inserted into the bone defect in order to evaluate physiological bone growth. For the Positive Control (+C), a Bio-Oss® bovine bone substitute (Geistlich Pharma AG, Wolhusen, Switzerland) was inserted into the bone defect. The amount of material introduced was approximately 7.0 mg. A biological membrane of bovine origin (GenDerm; Baumer, Mogi Mirim, São Paulo, Brazil) was placed under the Bio-Oss® to promote the isolated conduction of the bone substitute. For the Experimental group (EBS), approximately 7.0 mg of EBS were inserted into the bone defect and posteriorly covered by a biological membrane (GenDerm; Baumer, Mogi Mirim, São Paulo, Brazil) as described.

After surgical procedures, the periosteum was repositioned over the defect and sutured internally with polyglactin sutures (Catgut 4.0; Bioline, Anápolis, Goiás, Brazil), and the incision was closed with nylon sutures (Tecnofio 5.0; Anápolis, Goiás, Brazil). For analgesic purpose, animals received a dose of subcutaneous morphine (1 mL; Bayer, Germany) and antibiotic therapy with Cefalotina® (25 mg/kg; ABL, Cosmópolis, São Paulo, Brazil). During all experimental period, animals were housed in individual cages and maintained under a feeding regimen with rations and water *ad libitum* as well as good temperature, illumination and hygiene conditions. Euthanasia occurred in a humanized way using a CO₂ chamber (Insight—EB 248; Ribeirão Preto, São Paulo, Brazil) at 7, 21, 42 and 56 days postoperatively, being that six animals were sacrificed *per* group at each time-point.

The mandibles were removed with appropriate surgical technique, and the piece containing the bone defect was extracted. The pieces were fixed in 10% buffered formalin for 96 hours, decalcified in Morse solution for 30 days and thereafter submitted to routine laboratorial processing. The specimens were embedded in paraffin, and longitudinal 4,0-μm-thick sections were cut and stained with hematoxylin and eosin (H&E) for histological analysis.

### Histological and histomorphometric analysis

Histological analysis was performed using a digital camera with a resolution of 1.3 megapixels coupled to an optic microscope (Zeiss-Axio Imager 2, Oberkochen, Germany) with x5, x10, x20 and x40 magnifications. Additionally, images were analyzed using the software Image J (Jandel Scientific, San Rafael, CA, USA).

Semi-quantitative analysis about formation and quality of bone tissue were performed and scored as displayed in Table 1 [28]. For this, two blinded trained examiners evaluated each specimen independently. In the case of disagreement, the specimen was reevaluated, and a consensus was reached between examiners. For the histomorphometric evaluation, the original defect area was delimited and the metric calibration of the microscope was used as a standard. Within this region, the area of neoformed bone was measured and expressed as percentage. A same calibrated examiner performed all measurements. The reproducibility of results was tested by carrying out duplicate measurements in two samples form each group, at two different time points, one week apart. The results of the two recordings were then statistically analyzed with the Wilcoxon test for paired observations. No statistical significant differences were found between the two recordings (*p* < 0.05).

**Table 1:** Scores attributed to the formation and quality of bone tissue.

Score	Characterization
1	New tissue formation (filling of the defect with connective tissue containing blood capillaries, fibroblast, macrophage and newly formed collagen fibers).
2	Dense connective tissue suggesting bone tissue differentiation with the presence of a large number of cells and organized fibers.
3	New bone formation in which the connective tissue is differentiated to form a bone matrix or osteon.
4	Presence of bone tissue.

Adapted from Pretel H, Lizarelli RF, Ramalho LT. Effect of low-level laser therapy on bone repair: histological study in rats. Lasers Surg Med. 2007;39(10):788–796.

### Statistical analysis

Semi-quantitative scores were analyzed statistically through the Kruskal-Wallis test to compare the groups with respect to the formation and quality of bone tissue. Differences between histomorphometric data were analyzed through ANOVA two-way followed Tukey test. SAS 9.0 Software was used considering a significance level of 5%.

## Results

### Chemical characterization of EBS

The analysis of EBS by ICP-OES revealed a predominance of calcium in the composition, which was 71.68% of the total sample. Minor quantities of sodium and magnesium were also identified (less than 1% of total sample) (Table 2). Contaminant mineral materials did not reach significant values (Table 3).

**Table 2:** Expressed quantities of minerals in EBS detected by ICP-OES (g/kg).

Mineral	Symbol	g/kG
Calcium	Ca	5.52 ± 0.17
Potassium	K	0.019 ± 0.00032
Magnesium	Mg	0.517 ± 0.26
Manganese	Mn	0.005 ± 0.0004
Sodium	Na	1.429 ± 0.041
Phospor	P	0.211 ± 0.062
Iron	Fe	ND^a^
Zinc	Zn	ND^a^

^a^ Not detected.

**Table 3:** Expressed quantities of mineral contaminants in EBS detected by ICP-OES (g/kg).

Mineral	Symbol	g/kG
Aluminium	Al	0.004 ± 0.0038
Cadmium	Cd	ND^a^
Chrome	Cr	ND^a^
Copper	Cu	ND^a^
Mercury	Hg	0.0004 ± 0.0002
Pb Lead	Pb	0.011 ± 0.005

^a^ Not detected.

### Surface characterization of EBS and Bio-Oss®

**Fig 1:**
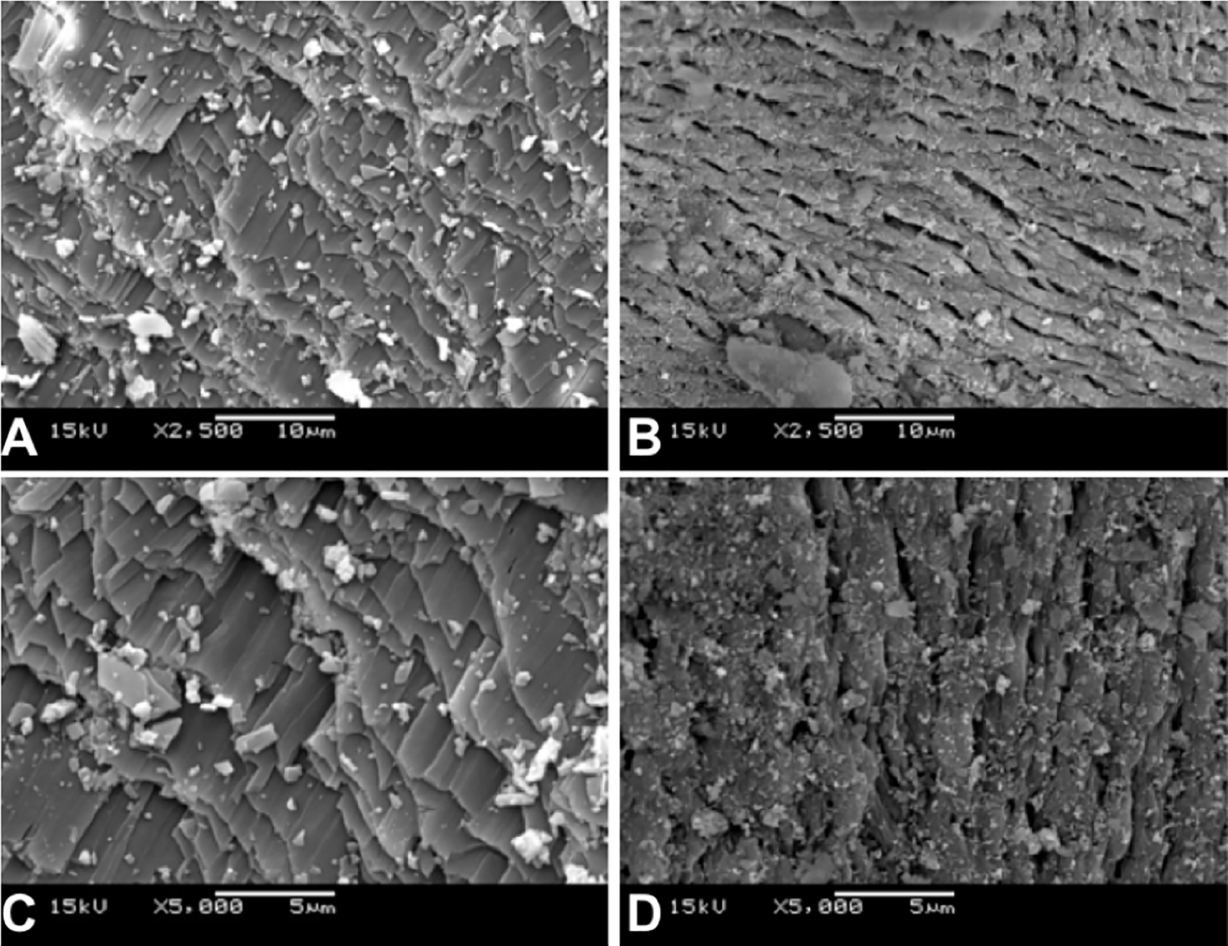
Surface characterization using SEM analysis of EBS at X2.500 (**A**) and X5.000 (**C**); and Bio-Oss® at X2.500 (**B**) and X5.000 (**D**).

The microstructure of EBS and Bio-Oss® was assessed using the SEM. It was observed in Fig 1A and 1C that EBS presents a larger heterogeneity in the particle size, while Bio-Oss® presented higher homogeneity (Fig 1B and 1D). Although EBS was prepared in a tightly controlled process, several morphological figures characterizing a porosity aspect were observed.

### Histological and Histomorphometric analysis

In the histological results, the absence of inflammatory process in the bone defects of all groups regardless of the time-point period was verified (i.e. 7, 21, 42 and 56 days). Considering the distribution of the animals according to the formation and quality of bone tissue, different scores according to the groups as well as progressive degrees of the bone neoformation process were detected, as detailed in Table 4. The area occupied by neoformed bone in each group is presented in Table 5. After 21 days of evaluation, the percentage of neoformed bone in the EBS group was 48.13 ± 21.15 of the original defect area, being significantly higher than–C and +C groups (*p* < 0.05). However, in the subsequent evaluation time-points (i.e. 42 and 56 days), it was not detected differences between groups (*p* > 0.05).

**Table 4:** Distribution of the animals according to the scores attributed to formation and quality of bone tissue.

		Scores^a^			
Groups	N^b^	1	2	3	4
**-C**					
7 days	6	6	0	0	0
21 days	6	2	4	0	0
42 days	6	0	2	4	0
56 days	6	0	0	2	4
**+C**					
7 days	6	6	0	0	0
21 days	6	0	6	0	0
42 days	6	0	0	4	2
56 days	6	0	0	2	4
**EBS**					
7 days	6	6	0	0	0
21 days	6	0	2	4	0
42 days	6	0	2	4	0
56 days	6	0	0	2	4

^a^ Scores according to Table 1.

^b^ Animal per group.

**Table 5:** Neoformed bone expressed as percentage of total defect area (mean ± SD).

		Neoformed bone area (%)		
Groups	N^a^	-C	+C	EBS
7 days	6	ND^b^	ND^b^	ND^b^
21 days	6	13.22 ± 7.15 (A,a)	11.08 ± 8.21 (A,a)	48.13 ± 21.15 (B,a)
42 days	6	59.17 ± 33.39 (A,b)	61.56 ± 29.12 (A,b)	68.07 ± 33.29 (A,b)
56 days	6	71.86 ± 31.09 (A,b)	69.21± 25.58 (A,b)	75.26 ± 35.11 (A,b)

^a^ Animal per group.

^b^ Not detected.

Different upper case letters indicates significant differences between groups at each time-point. Different lower case letters indicates significant differences between time-points in the same group. (ANOVA followed by Tukey test, *p* < 0.05).

#### Negative Control group (-C)

**Fig 2:**
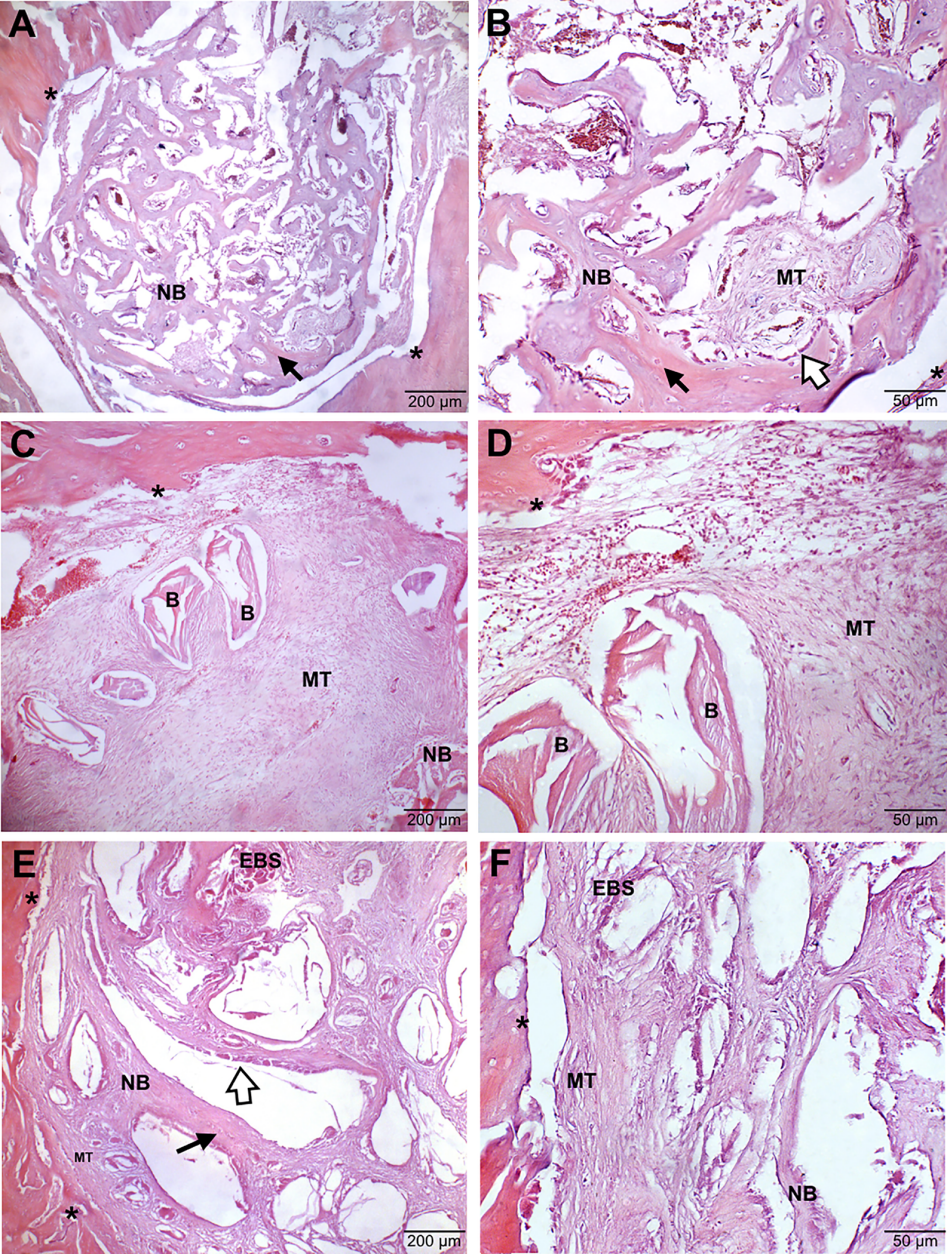
Histological findings of bone defect after 21 days. **A** and **B** represent the -C group with the presence of osteocytes (black arrow), osteoblasts (white arrow) and neoformed bone trabeculation (NB). **C** and **D** represent the +C group showing mesenchymal tissue (MT) and discret neoformed bone trabeculation (NB). **E** and **F** represent the EBS group where the predominance of MT and multifocal points of NB occupy the bone defect, presence of osteocytes and osteoblasts. B indicates Bio-Oss®; EBS indicates experimental bone substitute; * indicates the border of the bone defect. Tissues were stained with H&E and examined at 5x and 20x magnification (200 μm and 50 μm, repectively).

In the first 7 days, a predominance of disorganized connective tissue was verified, especially in the borders of the bone defect. Some cells with a cytological pattern compatible with mesenchymal/fibroblasts and osteoblasts were also observed. In subsequent time-point periods of evaluation (21 and 42 days), the neoformation progressed with the presence of osteocytes and neoformed bone trabeculation occupying the space once predominantly filled by connective tissue, characterizing an intramembranous ossification (Fig 2A and 2B). After 56 days, a progressive closure of the bone defect was visualized, presenting immature bone tissue with a lamellar patter containing osteoblasts and numerous osteocytes.

#### Positive Control group (+C)

This group demonstrated disorganized connective tissue in the first 7 days and bone differentiation after 21 days of evaluation, where mesenchymal/fibroblasts tissue and discrete neoformed bone trabeculation could be predominantly observed (Fig 2C and 2D). The process of bone neoformation was more pronounced at the borders of the bone defected but also occurring around the Bio-Oss®. After 56 days of evaluation, a significant intramembranous ossification was observed, with immature lamellar bone at the borders, multifocal points of border coaptation and a small region containing connective tissue in transformation at the center of the bone defect. The bone substitute used in this group (Bio-Oss®) persisted throughout all evaluation periods, being completely encased by connective tissue and/or neoformed trabecular bone.

#### Experimental group (EBS)

The histological progression pattern observed was close to the -C group. However, it is worth mentioning that, since the first 7 days, it was noted there was a large presence of mesenchymal/fibroblasts, osteoblasts and osteoclasts cells around the EBS, occupying the borders and center of the bone defect. Also, after 21 days, the predominance of mesenchymal/fibroblasts tissue and multifocal points of immature bone tissue almost totally occupying the area of the defect was already observed (Fig 2E and 2F). The neoformation and organization of the lamellar bone tissue and border cooptation in the 56 remaining days were more evident than in the -C group. Similar to Bio-Oss®, the EBS persisted throughout all evaluation periods, as it was completely encased by connective tissue and/or neoformed trabecular bone.

**Fig 3:**
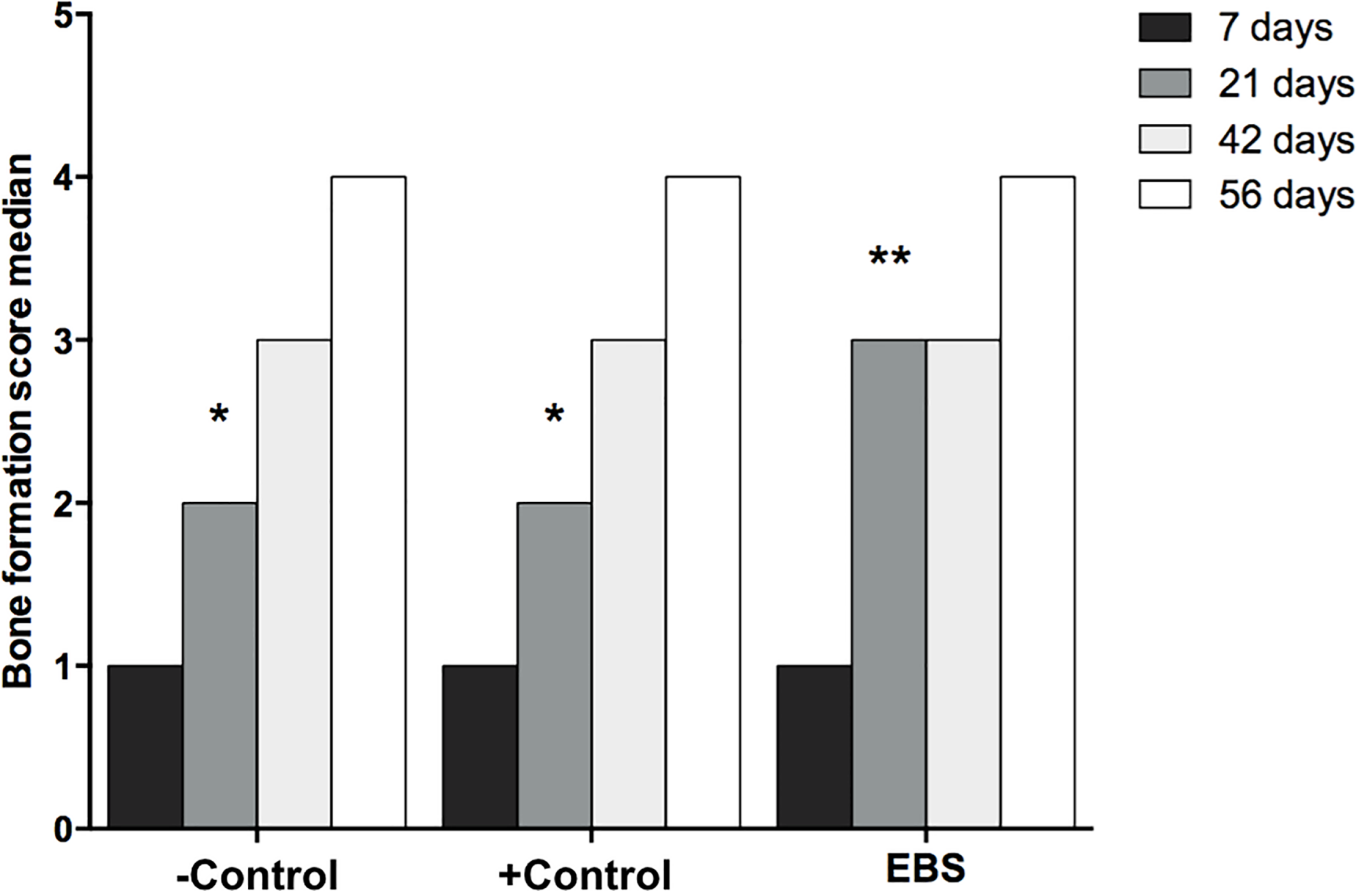
Bone formation score median between groups at different periods. Different symbols (*,**) indicate statistically significant differences between groups at the same period.

It was observed that the EBS group presented a more accelerated process of bone neoformation compared to the other groups, with a statistically significant difference between this group and the other groups in the 21-day time-point *(p* = 0.03112, Fig 3). However, after 42 days, the bone neoformation process was similar among all groups (*p* > 0.05), showing an immature lamellar bone pattern after 56 days of experimentation (*p* > 0.05).

## Discussion

An increase in tissue engineering has been noticed in the search for an ideal bone substitute [5–7]. Despite its demonstrated biocompatibility, biodegradability and osteogenic properties, nacre from oyster shells has been reported as a potential alternative to other commonly used materials for bone substitution [12,21–26]. Also, the use of oyster shells for bone grafting provides evidence with self-sustaining development that may reduce the cost for final rehabilitation treatment. Here we investigated the potential of a typical oyster shell of Northeastern Brazil (*C*. *rhizophora*) as a bone substitute.

The novelty of this study was demonstrated by the fast osteoinduction and osteogenesis stimulated by EBS using an animal model, which was described for the first time in the literature. The histological evidence indicates that the bone in EBS sites in the first time-points was denser when compared to a gold-standard bone substitute material (i.e. Bio-Oss®). Because neither bone-induction material nor stem cells or precursor cells were introduced at the EBS site, it may be possible that the release of active factors from nacre composition leads to the recruitment and differentiation of osteoprogenitor cells that give rise to bone cells and consequently produce new bone [21].

Nacre and human bone are both mineralized structures, although their composition and organization differ [4,8]. The mineral phase of bone is composed of calcium phosphate in the hydroxyapatite (HA) crystal form, whereas nacre is composed of calcium carbonate in an aragonite crystal form [27,29]. The chemical characterization confirmed that the main element found in the EBS was calcium (Ca; 71.68% of total sample), which is in accordance with several studies [23–26,29–34]. However, the composition of our samples differed from that of Western Mediterranean oyster samples (particularly the most studied oyster, the giant *Pinctada maxima* [22–26,31–36]), indicating a high variation of these components according to the geographic origin. Also, the amounts of calcium detected were relatively higher than described elsewhere [23,29,30], thus probably being one of the factors responsible for the good histological results.

It is known that the most used osteoconductive scaffolds are calcium-sulfate and calcium-phosphate substitutes, since they allow the growth of capillaries, perivascular tissue and mesenchymal stem cells, forming a structure like that of spongy bone [31]. However, in addition to serving as an osteoconduction scaffold, the EBS histological results demonstrated a progression pattern close to that observed in the -C group (i.e. physiological bone growth). This result suggests that, in addition to osteoconduction, EBS was able to induce osteogenesis, which leads us to believe in its potential osteoinduction. Calcium substitutes also share several characteristics that make them a good choice: slow biodegradation, superior strength in compression and unique capacity for osseointegration, or the ability of the bone’s host to interdigitate with a rough crystalline graft interface [26,32].

Additionally, SEM analyses revealed that EBS presented larger heterogeneity in the particle size compared to the Bio-Oss® group, and its composition resembles a cluster of other sub-particles characterizing a porosity aspect. It is known that the structural characteristics of a scaffold for tissue engineering directly affect the cellular response and must be elaborated on to promote cell adhesion, proliferation and differentiation [17]. It is possible that the microporosity present in EBS guarantees high capillary action, an ideal environment for bone formation, as it allows the entry of blood, osteoprogenitor cells and proteins into the particles of the material.

Early studies [26,32] investigated the potential application of the powder of *P*. *maxima* to bone grafting using animal models. Their results showed newly formed bone in close contact with the nacre placement, in that the nacre particles in contact with the trabeculae gradually dissolved after 56 days. Our findings are in agreement, where the presence of osteoblasts and osteoclasts around the EBS from the first 7 days of evaluation and initial bone formation in only 21 days was observed, which was confirmed by histological and histomorphometric data. Compared to control groups, this bone formation it was only expressive after 42 days. Also, EBS showed good biocompatibility to bone that was confirmed for the absence of inflammatory process and border cooptation in bone defects regardless of the time-point period.

Regarding the union of the EBS particles with the bone, it was verified that, after 56 days, there were still points of cooptation of the newly formed bone with the edges of the bone defect. This “cement line” may represent not only a chemical union of the bone with the EBS but also a micromechanical interdigitating possibly of non-collagenous proteins, which is a favorable signal for the osteoclast migration and bone remodeling. It was previously observed [33] that the use of solid nacre was bonded directly to the newly formed bone, without interference by cells, which was different from our findings. This may be due to our use of nacre powder instead of solid nacre.

An investigation about the mineral and organic phases of *P*. *maxima* revealed the presence of a water-soluble matrix (WSM) existing at the organic-mineral interface of the nacres layer [34,35]. These studies demonstrated the tight linkage of the matrix proteins and the mineral phase. It is also suggested that the WSM presents BMPs, which induce bone growth [27,32] and had a great effect on MC3T3 pre-osteoblast cells, accelerating differentiation and mineralization [36]. Studies using mammalian bone marrow cells observed that WSM induces rapid mineralization and that fibroblasts displayed an early increase in alkaline phosphatase (ALP) activity [35–38]. These results indicated the osteogenic potential of nacre, and it is possible that the greater the mineral availability, the better this organic-mineral interface formed. The literature has never documented an oyster that presents higher amounts of Ca such as *C*. *rhizophora*, which could suggest the formation of a layer responsible for great potential of bioactivity.

The results of this initial study indicate that the nacre particles from *C*. *rhizophora* can promote bone formation more rapidly than a gold-standard bone substitute material. In fact, after 21 days, the bone formation in the EBS occurs in a similar way to the other groups, which leads us to believe that the mechanisms of osteoinduction happen incisively in the early stages of bone formation. Thus, future studies into the mineral and organic contents of *C*. *rhizophora* will hopefully reveal the regulatory mechanisms of osteogenesis that will optimize the possible use of this material in tissue engineering. Added to the high calcium contents detected, it is hypothesized that the use of nacre as an alternative bone-substitute material appears to be promising.

## Conclusion

Within the limitations of this first study, it was possible to conclude that EBS presented good biocompatibility and promoted fast stimulation for bone-forming cells in an animal model.

## Supporting information

S1 Fig Surgical procedure details: (**A)** After shaving and asepsis of the submandibular area, an incision was made and skin and periosteal flaps were elevated. (**B**) Bone defect was prepared with a cylindrical stainless-steel bur (3 mm diameter; 2 mm deep). (**C**) Negative Control group, where no bone substitutes were inserted. (**D**) Positive Control group, where Bio-Oss® was inserted and protected by a biological membrane. (**E**) Experimental group, where EBS was inserted and protected by a biological membrane. (**F**) Aspect after suture of all groups tested.
(JPEG)

## References

[1] Lang NP, Berglundh T, Heitz-Mayfield LJ, Pjetursson BE, Salvi GE, Sanz M. Consensus statements and recommended clinical procedures regarding implant survival and complications. Int J Oral Maxillofac Implants. 2004;19(suppl):150–154. 15635955

[2] Misch CE, Perel ML, Wang HL, Sammartino G, Galindo-Moreno P, Trisi P, et al Implant success, survival, and failure: the international congress of oral implantologists (ICOI) Pisa consensus conference. Implant Dent. 2008;17:5–15. doi: 10.1097/ID.0b013e3181676059 18332753 10.1097/ID.0b013e3181676059

[3] Penarrocha-Diago M, Penarrocha-Diago M, Zaragozí-Alonso R, Soto-Penaloza D. Consensus statements and clinical recommendations on treatment indications, surgical procedures, prosthetic protocols and complications following all-on-4 standard treatment. J Clin Exp Dent. 2017;9(5):e712–715. doi: 10.4317/jced.53759 28512551 10.4317/jced.53759 PMC5429486

[4] Chiapasco M, Zaniboni M, Boisco M. Augmentation procedures for the rehabilitation of deficient edentulous ridges with oral implants. Clin Oral Implants Res. 2006;17(suppl 2):136–159. 16968389 10.1111/j.1600-0501.2006.01357.x

[5] Buser D, Dula K, Hirt HP. Lateral ridge augmentation using auto-grafts and barrier membranes: a clinical study with 40 partially edentulous patients. J Oral Maxillofac Surg. 1996; 54:420–432. 8600258 10.1016/s0278-2391(96)90113-5

[6] Schwartz-Arad D, Levin L, Sigal L. Surgical success of intraoral autogenous block onlay bone grafting for alveolar ridge augmentation. Implant Dent. 2005; 2:131–138. 10.1097/01.id.0000165031.33190.0d 15968184

[7] Lohmann H, Grass G, Rangger C, Mathiak G. Economic impact of cancellous bone grafting in trauma surgery. Arch Orthop Trauma Surg. 2007;127:345–348. doi: 10.1007/s00402-006-0277-4 17294203 10.1007/s00402-006-0277-4

[8] Sakkas A, Wilde F, Heufelder M, Winter K, Schramm A. Autogenous bone grafts in oral implantology-is it still a “gold standard”? A consecutive review of 279 patients with 456 clinical procedures. Int J Implant Dent. 2017;3(1):23 doi: 10.1186/s40729-017-0084-4 28573552 10.1186/s40729-017-0084-4 PMC5453915

[9] Lye KW, Deatherage JR, Waite PD. The use of demineralized bone matrix for grafting during Le Fort I and chin osteotomies: techniques and complications. J Oral Maxillofac Surg. 2008;66:1580–1585. doi: 10.1016/j.joms.2007.12.003 18634943 10.1016/j.joms.2007.12.003

[10] Raghoebar GM, Louwerse C, Kalk WW, Vissink A. Morbidity of chin harvesting. Clin Oral Implants Res. 2001;12:503–507. 11564111 10.1034/j.1600-0501.2001.120511.x

[11] Joshi A. An investigation of post-operative morbidity following chin graft surgery. Br Dent J. 2004;196(4):215–218. doi: 10.1038/sj.bdj.4810987 15039731 10.1038/sj.bdj.4810987

[12] Bahar H, Yaffe A, Binderman I. The influence of nacre surface and its modification on bone apposition: a bone development model in rats. J Periodontol. 2003;74(3):366–371. doi: 10.1902/jop.2003.74.3.366 12710757 10.1902/jop.2003.74.3.366

[13] Goldberg VM. Selection of bone grafts for revision total hip arthroplasty. Clin Orthop Relat Res. 2000;(381):68–76. 11127672 10.1097/00003086-200012000-00008

[14] Perry CR. Bone repair techniques, bone graft, and bone graft substitutes. Clin Orthop Relat Res. 1999;(360):71–86. 10101312 10.1097/00003086-199903000-00010

[15] Tay BK, Patel VV, Bradford DS. Calcium sulfate- and calcium phosphate-based bone substitutes. Mimicry of the mineral phase of bone. Orthop Clin North Am. 1999;30(4):615–623. 10471766 10.1016/s0030-5898(05)70114-0

[16] Kasten P, Luginbuhi R, Van Griensven M, Barkhausen T, Krettek C, Bohner M, et al Comparison of human bone marrow stromal cell seeded on calcium-deficient hydroxyapatite, b-tricalcium phosphate and demineralized bone matrix. Biomaterials. 2003;24:2593–2603. 12726713 10.1016/s0142-9612(03)00062-0

[17] Schwartz-Arad D, Levin L, Sigal L. Surgical success of intraoral autogenous block onlay bone grafting for alveolar ridge augmentation. Implant Dent. 2005;2:131–138. 10.1097/01.id.0000165031.33190.0d 15968184

[18] Li H, Pujic Z, Bartold PM. Identification of bone morphogenetic protein 2 and 4 in commercial demineralized freeze-dried bone allograft preparations: pilot study. Clin Implant Dent Relat Res. 2000; 2:110–117. 11359264 10.1111/j.1708-8208.2000.tb00113.x

[19] Boyan B, Ranly DM, McMillan J, Sunwoo M, Roche K, Schwarts Z. Osteoinductive ability of human allograft formulations. J Periodontol. 2006;77:1555–1563. doi: 10.1902/jop.2006.060019 16945034 10.1902/jop.2006.060019

[20] Harris CT, Cooper LF. Comparison of bone graft matrices for human mesenchymal stem cell directed osteogenesis. J Biomed Mater Res. 2004; 68:747–755. 10.1002/jbm.a.20107 14986329

[21] Asvanund P, Chunhabundit P. Alveolar bone regeneration by implantation of nacre and B-tricalcium phosphate in guinea pig. Implant Dent. 2012;21(3):248–253. doi: 10.1097/ID.0b013e3182563ae0 22614846 10.1097/ID.0b013e3182563ae0

[22] Mouries LP, Almeida MJ, Milet C, Berland S, Lopez E. Bioactivity of nacre water-soluble organic matrix from the bivalve mollusk *Pinctada maxima* in three mammalian cell types: fibroblasts, bone marrow stromal cells and osteoblasts. Comp Biochem Physiol B Biochem Mol Biol. 2002;132:217–229. 11997223 10.1016/s1096-4959(01)00524-3

[23] Hamza S, Bouchemi M, Slimane N, Azari Z. Physical and chemical characterization of adsorbed protein onto gold electrode functionalized with Tunisian coral and nacre. Mater Sci Eng C Mater Biol Appl. 2013; 33(1):537–542. doi: 10.1016/j.msec.2012.09.028 25428107 10.1016/j.msec.2012.09.028

[24] Flausse C, Henrionnet M, Dossot D, Dumas S, Hupont A, Pinzano D, et al Osteogenic differentiation of human bone marrow mesenchymal stem cells in hydrogel containing nacre powder. J Biomed Mater Res, Part A. 2013;101:3211–3218. 10.1002/jbm.a.34629 23554327

[25] Zhang G, Brion A, Willemin AS, Piet MH, Moby V, Bianchi A, et al Nacre, a natural, multi-use, and timely biomaterial for bone graft substitution. J Biomed Mater Res A. 2017;105(2):662–671. doi: 10.1002/jbm.a.35939 27750380 10.1002/jbm.a.35939

[26] Gerhard EM, Wang W, Li C, Guo J, Ozbolat IT, Rahn KM, et al Design strategies and applications of nacre-based biomaterials. Acta Biomater. 2017;54:21–34. doi: 10.1016/j.actbio.2017.03.003 28274766 10.1016/j.actbio.2017.03.003

[27] Miyashita T, Hanashita T, Toriyama M, Takagi R, Akashika T, Higashikubo N. Gene cloning and biochemical characterization of the BMP-2 of *Pinctada fucata*. Biosci Biotechnol Biochem. 2008;72(1):37–47. 18175919 10.1271/bbb.70302

[28] Pretel H, Lizarelli RF, Ramalho LT. Effect of low-level laser therapy on bone repair: histological study in rats. Lasers Surg Med. 2007;39(10):788–796. doi: 10.1002/lsm.20585 18081142 10.1002/lsm.20585

[29] Ni M, Ratner BD. Nacre surface transformation to hydroxyapatite in a phosphate buffer solution. Biomaterials. 2003;24:4323–4331. 12853263 10.1016/s0142-9612(03)00236-9

[30] Shen Y, Zhu J, Zhang H, Zhao H, Zhao F. In vitro osteogenetic activity of pearl. Biomaterials. 2006;27(2):281–287. doi: 10.1016/j.biomaterials.2005.05.088 16023711 10.1016/j.biomaterials.2005.05.088

[31] Sciadini MF, Dawson JM, Johnson KD. Evaluation of bovine-derived bone protein with a natural coral carrier as a bone-graft substitute in a canine segmental defect model. J Orthop Res. 1997;15(6):844–857. doi: 10.1002/jor.1100150609 9497809 10.1002/jor.1100150609

[32] Lamghari M, Almeida MJ, Berland S, Huet H, Laurent A, Milet C, et al Stimulation of bone marrow cells and bone formation by nacre: in vivo and in vitro studies. Bone. 1999;25:91S–94S. 10458284 10.1016/s8756-3282(99)00141-6

[33] Liao H, Mutvei H, Hammarstrom L, Wurtz T, Li J. Tissue responses to nacreous implant in rat femur: an in situ hybridization and histochemical study. Biomaterials. 2002;23:2693–2701. 12059018 10.1016/s0142-9612(01)00421-5

[34] Balmain J, Hannoyer B, Lopez E. Fourier transform infrared spectroscopy (FTIR) and X-ray diffraction analyses of mineral and organic matrix during heating of mother of pearl (nacre) from the shell of the mollusc *Pinctada maxima*. J Biomed Mater Res. 1999;48:749–754. 10490692 10.1002/(sici)1097-4636(1999)48:5<749::aid-jbm22>3.0.co;2-p

[35] Almeida MJ, Milet C, Peduzzi J, Pereira L, Haigle J, Barthelemy M, et al Effect of water-soluble matrix fraction extracted from the nacre of *Pinctada maxima* on the alkaline phosphatase activity of cultured fibroblasts. J Exp Zool. 2000;288:327–334. doi: 10.1002/1097-010X(20001215)288:4<327::AID-JEZ5>3.0.CO;2-# 11144281 10.1002/1097-010X(20001215)288:4<327::AID-JEZ5>3.0.CO;2-#

[36] Rousseau M, Pereira-Mouriès L, Almeida MJ, Milet C, Lopez E. The water-soluble matrix fraction from the nacre of *Pinctada maxima* produces earlier mineralization of MC3T3-E1 mouse pre-osteoblasts. Comp Biochem Physiol B: Biochem Mol Biol. 2003;135:1–7. 12781967

[37] Milet S, Berland M, Lamghari L, Mouries C, Jolly C, Borzeix S, et al Conservation of signal molecules involved in biomineralisation control in calcifying matrices of bone and shell. C R Palevol.2004;3:493–501.

[38] Westbroek P, Marin F. A marriage of bone and nacre.Nature.1998;392:861–862. doi: 10.1038/31798 9582064 10.1038/31798

