# Supplementary figures and images for: Bone substitute made from a Brazilian oyster shell functions as a fast stimulator for bone-forming cells in an animal model
Source: PLoS One. 2018 Jun 5;13(6):e0198697. doi: 10.1371/journal.pone.0198697 (PMC5988300; doi:10.1371/journal.pone.0198697)

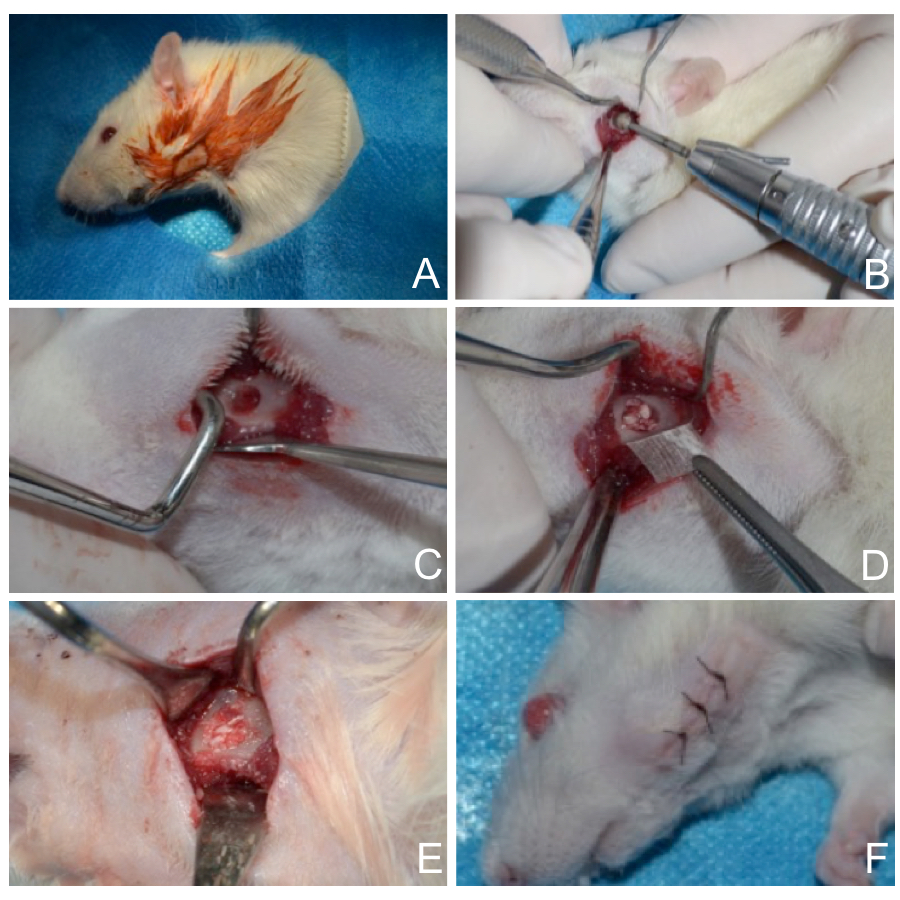

Supplement: S1 Fig — Surgical procedure details: (A) After shaving and asepsis of the submandibular area, an incision was made and skin and periosteal flaps were elevated. (B) Bone defect was prepared with a cylindrical stainless-steel bur (3 mm diameter; 2 mm deep). (C) Negative Control group, where no bone substitutes were inserted. (D) Positive Control group, where Bio-Oss® was inserted and protected by a biological membrane. (E) Experimental group, where EBS was inserted and protected by a biological membrane. (F) Aspect after suture of all groups tested. (JPEG) [file pone.0198697.s001.jpeg]
